# Kaposi’s sarcoma: a 10-year experience with 248 patients at a single tertiary care hospital in Tanzania

**DOI:** 10.1186/s13104-015-1348-9

**Published:** 2015-09-15

**Authors:** Phillipo L. Chalya, Fidelis Mbunda, Peter F. Rambau, Hyasinta Jaka, Nestory Masalu, Mariam Mirambo, Martha F. Mushi, Samuel E. Kalluvya

**Affiliations:** 1 Department of Surgery, Bugando Medical Centre, Mwanza, Tanzania; 2 Department of Pathology, Catholic University of Health and Allied Science-Bugando, Mwanza, Tanzania; 3 Department of Internal Medicine, Catholic University of Health and Allied Science-Bugando, Mwanza, Tanzania; 4 Department of Oncology, Bugando Medical Centre, Mwanza, Tanzania; 5 Department of Microbiology, Catholic University of Health and Allied Science-Bugando, Mwanza, Tanzania

**Keywords:** Kaposi’s sarcoma, Human immunodeficiency virus, Acquired immunodeficiency syndrome, Clinicopathological, Treatment outcome, Tanzania

## Abstract

**Background:**

Kaposi’s Sarcoma is the most common sarcoma and second most prevalent cancer seen in Tanzania. Little is known about Kaposi’s sarcoma in our setting as there is paucity of recent published data regarding this condition. This study describes the clinicopathological pattern and treatment outcome of Kaposi’s sarcoma at Bugando Medical Centre, a tertiary care hospital in northwestern Tanzania.

**Methods:**

This was a prospective study of histologically confirmed Kaposi’s sarcoma that was conducted at Bugando Medical Center between July 2004 and June 2014.

**Results:**

A total of 248 patients (M:F = 1.4:1) representing 2.4 % of all malignancies during the study period were enrolled into the study. The median age at presentation was 36 years. Females were younger than males (p = 0.04). Out of 248 patients, 122 (49.2 %) were HIV positive. Of these, 46 (37.7 %) were males and 76 (62.3 %) females. AIDS-related Kaposi’s sarcoma were younger than HIV negative Kaposi’s sarcoma patients (p = 0.011). Median duration of symptoms was 6 months. Kaposi’s sarcoma was the AIDS defining disease in 82 (67.2 %) patients while in the remaining 40 (32.8 %) it was diagnosed between 1 and 14 months after the initial diagnosis of AIDS. The lower limb was most frequently involved site in 28.9 % of patient. Females had more disseminated lesions compared with more localized lesions in the males (p = 0.001). The treatment modalities in this study included chemotherapy, radiotherapy, surgery and highly active antiretroviral therapy. Overall 126 (53.4 %) patients had significant improvement in quality of life at the end of 1 year follow up. Treatment related complication and mortality rates were 25.8 and 24.2 % respectively. Poor ACTG stage, CD4+ count <200 cells/µl, associated co-morbid illness, disseminated disease and poor adherent to chemotherapy were the significant independent factors associated with deaths (p < 0.001). Patient’s follow-up was generally poor and data on long-term survivals were not available as more than two-thirds of patients were lost to follow up.

**Conclusion:**

Kaposi’s sarcoma is the most common malignant vascular tumor and HIV/AIDS- related cancer in our region. There is an urgent need to develop health education programmes to enhance the understanding of this disease and how it spreads, particularly among the younger generation.

## Background

Kaposi’s sarcoma (KS) is a malignant neoplasm of the vascular endothelium that is multifocal in origin involving the skin and other organs [1]. It was first described in 1872 by Moritz Kaposi, a Hungarian dermatologist who described KS as a rare multifocal angioproliferative tumor involving blood and lymphatic vessels and it was thought to occur only in Eastern Europe and the Mediterranean [2, 3]. However, Kaposi’s sarcoma is now the most frequently reported malignant skin tumor in some areas of Africa and was endemic in Africa even before the advent of the human immunodeficiency virus (HIV) [4, 5]. Kaposisi’s sarcoma has become the most common neoplasm in AIDS patients since it was first reported among homosexual men in the United States at the beginning of the AIDS epidemic [6]. On the basis of clinical and epidemiological features, four types of Kaposi’s sarcoma have been recognized: classic, endemic (African), iatrogenic and epidemic (AIDS related). The course of Kaposi’s sarcoma ranges from indolent, with only skin manifestations to fulminant with extensive visceral involvement [7].

Kaposi’s sarcoma poses problems in a histologic diagnosis because of its broad morphologic spectrum and similarity to many benign vasoproliferative lesions (e.g. pyogenic granuloma, bacillary angiomatosis, and microvenular hemangioma) and tumors with a prominent spindle cell component (e.g. spindle cell hemangioma, spindle cell angiosarcoma, and dermatofibrosarcoma protuberans) [8, 9]. The variability in the biologic behavior of Kaposi’s sarcoma among individuals in the same group remains enigmatic.

Since the emergence of human immunodeficiency virus (HIV) infection, there has been a steady increase in the prevalence of Kaposi’s sarcoma worldwide. In the past, the incidence of Kaposi’s sarcoma was over 20,000 times higher in patients with AIDS than in the general population [10]. However, the discovery and widespread use of highly active antiretroviral therapy (HAART) in developed countries has led to a substantial decrease in the incidence of AIDS-related Kaposi’s sarcoma (AIDS-KS). In addition, patients on HAART present with less aggressive disease which is associated with less morbidity and mortality [11]. The burden of HIV infection and AIDS is greatest in the developing world and neoplastic complications are increasingly encountered [12]. Although Kaposi’s sarcoma was endemic in central and east Africa before the AIDS epidemic, AIDS- Kaposi’s sarcoma has become the most frequently diagnosed tumor in several African countries [13–15]. The incidence has been steadily increasing partly because of limited access to antiretroviral drugs and other preventive or curative therapies for AIDS associated cancers [15].

Kaposi’s sarcoma affects all ages, but lymph node involvement is more frequent in children and adolescents. The disease predominantly affects men (M:F of 12:1–3:1) [16]. The lower limbs are reported to be the predominant site affected in Africans [13–16].

The pathogenesis of Kaposi’s sarcoma is largely obscure and has been a subject of debate. In 1994, Chang et al. [17] discovered HHV-8, or the Kaposi’s sarcoma-associated herpes virus, as the causative agent of acquired immunodeficiency syndrome associated Kaposi’s sarcoma and it has subsequently been found in all epidemiologic forms. Subsequent studies have showed that in over 95 % of Kaposi’s sarcoma, KSHV is present regardless of HIV status [16, 18, 19]. It has now been established that KSHV is the primary and necessary factor in the development of Kaposi’s sarcoma [4, 16].

Although the incidence of AIDS-related Kaposi’s sarcoma is reported to have fallen by more than 90 % in some populations with the introduction of highly active antiretroviral therapy (HAART) and safe sexual practices, the situation may be the reverse in most of Africa due not only to a more rapid rate of progression of HIV disease but also to the unavailability of HAART in most communities [20].

Kaposi’s sarcoma is the most common malignant vascular tumor and second most prevalent cancer seen in Tanzania and is commonly associated with HIV/AIDS [21–23]. Most patients present late after prior traditional medical treatment. However, despite this observation, there is a paucity of information regarding this condition in Tanzania and particularly the study area. This is partly due to a lack of published local data regarding Kaposi’s sarcoma and lack of cancer registries in this region. This study was designed to describe our ten-year experience in the management of Kaposi’s sarcoma outlining the clinicopathological pattern and treatment outcome of Kaposi’s sarcoma at Bugando Medical Centre, a tertiary care hospital in northwestern Tanzania.

## Methods

### Study design and setting

Between July 2004 and June 2014, a prospective study of histologically confirmed cases of Kaposi’s sarcoma was conducted at Bugando Medical Center. Bugando Medical Center is a tertiary care and teaching hospital for the Catholic University of Health and Allied Sciences-Bugando (CUHAS-Bugando) in the Lake and Western Zones of the United Republic of Tanzania. It has 1000 beds and serves as a referral center for tertiary specialist care for a catchment population of approximately 13 million people.

### Study population

The study included all histologically confirmed cases of Kaposi’s sarcoma seen at Bugando Medical Center during the period of study. Patients who had no histological results and those who refused to test for HIV infection were excluded from the study. The sample size for the study was determined using the Kish and Lisle formula (1965) as follows: n = Z^2^ p (1 − p)/d2 where, Zα = value at a specified confidence level, p = approximate proportion of the event in the population and d = acceptable margin of error in estimating the true population proportion. The proportion of patients with Kaposi’s sarcoma in Tanzania is not known and it was estimated at 50 %, and given 1.96 value of the 95 % confidence interval and 0.05 an acceptable margin of error, the minimum sample size required for the study was:

$$\begin{aligned} {\text{n}}&= \frac{ 1.96\times  1.96  \times  0.50\left(1-0.50 \right)}{0.05 \times 0.05} \hfill \\ =  384 \hfill \\ \end{aligned}$$

The minimum sample size required for the study therefore was 384 patients. However, in this study only 248 patients were available for the final analysis. Patients were screened for inclusion criteria and those who met the inclusion criteria were offered explanations about the study and requested to consent before being enrolled into the study. The diagnosis of Kaposi’s sarcoma was made clinically and confirmed histologically. Patients were assessed at the time of diagnosis of Kaposi’s sarcoma. The weight, vital signs and Kaposi’s sarcoma symptoms including pain, limb swelling, ulceration and disfigurement were recorded, and a physical examination was performed. The tumor (T) size was defined as the sum of the greatest diameters of each measurable tumor. All patients in this study were requested to test for HIV infection using Tanzania HIV Rapid Test Algorithm [24] and CD4+ count using FACS or FACSCALIBUR from BD Biosciences USA. A determination of CD4 count was only performed in HIV positive patients. In patients with AIDS-related Kaposi’s sarcoma, the Karnofsky score status of the patients was recorded. The tumor was staged according to AIDS Clinical Trials Group (ACTG) classification using the tumor (T), immune system (I) and systemic illness (S) [25]. Tumor was defined as T_0_ (localized disease) if the disease was confined to the skin and lymph nodes or oral involvement was confined to the hard palate, or T1 (disseminated disease) if there was pulmonary or gastrointestinal involvement, tumor associated edema or ulceration, or extensive oral involvement. Morphologically the lesions were classified as macular, nodular and ulcerative. I (Immune system status): I_0_ (good risk): CD4 cell count >200 cells/µl; I_1_ (poor risk): CD4 cell count <200 cells/µl. S (Systemic illness status): S_0_ (good risk): no systemic illness present; S_1_ (poor risk): systemic illness present with one or more of the following: Presence of opportunistic infection or thrush; One or more of B symptoms e.g. fever or night sweats; Performance status score less than 70 (Karnofsky performance status score); Other HIV related illness present e.g. neurological disease or lymphoma.

In every patient, biopsied tissue from representative Kaposi’s sarcoma lesion was fixed in 10 % formalin and embedded in paraffin wax. Routine histological sections were prepared using standard staining methods with H and E. The histologic features of Kaposi’s sarcoma included spindle-shaped tumor cells surrounding hyperemic vascular slits, often accompanied by extravasated erythrocytes, hemosiderin, and fibrosis.

Serum urea and electrolytes, liver function test and complete blood count with differential and platelet count were done at the time of diagnosis of Kaposi’s sarcoma and before administration of chemotherapy. Chest radiographs, ECG, abdominal ultrasound, faecal occult blood and gastrointestinal endoscopic examination were performed in appropriate cases. All patients were treated according to Bugando Medical Centre protocol which recommends that patients with Kaposi’s sarcoma are started with Anti-KS therapy. This consisted of six courses of three weekly cycles of either vincristine monotherapy or as a combination of vincristine, doxorubicin and bleomycin. In addition to Anti- Kaposi’s sarcoma chemotherapy, patients with AIDS-related Kaposi’s sarcoma should start on HAART irrespective of CD4+ count. Radiotherapy and surgical excision of localized lesions were performed in appropriate cases. Treatment complications and outcome were monitored. Data on each patient were entered into a questionnaire prepared for the study. The study variables included age of patients at diagnosis, sex, HIV status, associated co-morbid conditions, duration of illness, clinical presentation, anatomical sites, tumor stage (ACTG), CD4+ count, treatment offered, treatment related complications and mortality. Follow-up of patients was for 12 months with documentation of outcome at the end of the observation period.

### Statistical data analysis

The statistical analysis was performed using the Statistical Package for Social Sciences (SPSS) version 17.0 for Windows (SPSS, Chicago, Illinois, USA). The median (and IQR) and ranges were calculated for continuous variables, whereas proportions and frequency tables were used to summarize categorical variables. The Chi square (χ^2^) test was used to test for the significance of association between the independent (predictor) and dependent (outcome) variables in the categorical variables. Student *t* test was used to test for significance of associations between the predictor and outcome variables in the continuous variables. The level of significance was considered as P < 0.05. Multivariate logistic regression analysis was used to determine predictor variables that predicted the outcome.

### Ethical consideration

Ethical approval to conduct the study was obtained from the CUHAS-Bugando/BMC joint institutional ethic review committee before the commencement of the study. Informed consent was sought from each patient before being enrolled into the study.

## Results

### Patient’s characteristics

During the study period, a total of 10324 malignancies were registered at Bugando Medical Centre. Of these, 258 (2.4 %) were histopathologically confirmed Kaposi’s sarcoma giving an average of 25 cases annually. Out of these, 10 patients were excluded from the study due to missing data. Thus, 248 patients were enrolled into the study. The age of patients at diagnosis ranged from 3 to 76 years with a median of 36 years (IQR = 34–38 years). The modal age group was 31–40 years. There were 144 (58.1 %) males and 104 (41.9 %) females with a male to female ratio of 1.4: 1. Females were younger than males (median age of 28 years vs. males 37; p = 0.04). Out of 248 patients, 122 (49.2 %) were HIV positive. Of these, 46 (37.7 %) were males and 76 (62.3 %) females. Their ages ranged from 5 to 54 years with a median of 28 years (IQR = 26–32 years). AIDS-related Kaposi’s sarcoma were younger than HIV negative KS patients (median age of 26 years among AIDS-KS vs median age of 48 years among HIV seronegative KS) and this was statistically significant (p = 0.011). Co-morbid conditions were seen in 73 (29.4 %) patients including 28 (38.4 %) pulmonary tuberculosis, 18 (24.7 %) anorectal sepsis, 12 (16.4 %) oral candidiasis, 6 (8.2 %) hypertension, 5 (6.6 %) diabetes mellitus and 4 (5.5 %) peptic ulcers. Co-morbid conditions were more common in patients with AIDS-related Kaposi’s sarcoma than in those without AIDS-KS (62/122 = 46.6 versus 11/126 = 8.7 %). This difference was statistically significant (p = 0.011).

### Clinicopathological presentation

The duration of symptoms of Kaposi’s sarcoma ranged from 1 to 14 months with a median of 6 months (IQR = 4–8 months). Symptoms of Kaposi’s sarcoma were present in 212 (85.5 %) patients. The most common symptoms were swelling of extremities 124 (58.5 %), pain in 98 (46.2 %) and cosmetic disabilities in 52 (25.5 %) patients. Thirty-six (14.5 %) patients had no symptoms of Kaposi’s sarcoma and the diagnosis was made during routine clinical evaluation of other diseases. Amongst the 122 HIV patients, Kaposi’s sarcoma was the AIDS defining disease in 82 (67.2 %) patients while in the remaining 40 (32.8 %) it was diagnosed between 1 and 15 months after the initial diagnosis of AIDS. Twenty-two patients had used HAART for 1–13 months at the time of diagnosis of AIDS-related Kaposi’s sarcoma. Most of HIV patients, 84 (68.9 %) had Karnofsky performance score ≥70 at the time of diagnosis. A total of 387 anatomical sites were documented in 248 patients giving an average of 1.6 anatomical sites per patients. The majority patients, 214 (86.3 %) had multiple lesions (disseminated disease) and only 34 (13.7 %) had isolated lesions (localized disease). AIDS-related Kaposi’s sarcoma patients were more likely to present with disseminated form of the disease as compared to HIV seronegative patients (67.7 vs 26.1 %, p = 0.014). At the time of diagnosis females had more widespread and advanced AIDS- related Kaposi’s sarcoma compared to males (45.9 vs 18.6 %, p = 0.003). Skin 241 (62.3 %) was most primary organ affected followed by oral cavity in 54 (14.0 %). The lower limb was most frequently involved anatomical site in 28.9 % of patients (Table 1). Visceral lesions included 4 rectal, 6 intestinal and 2 gastric tumors and accounted for 3.1 % of patients. Lymph node involvement in patients with AIDS-KS commonly occurred in adults whereas in patients without AIDS-related Kaposi’s sarcoma lymph node involvement was exclusively a disease of children. A total of 148 (69.8 %) patients were found to be anemic. Out of these, 118 (79.7 %) had AIDS-KS and the remaining 30 (20.3 %) patients had KS without HIV/AIDS. Tumor size ranged from 2 to 64 cm with a median of 32 cm (IQR = 28 to 34 cm). The histological type was mixed cellularity in 162 (63.3 %) patients, monomorphic in 56 (22.6 %) patients and anaplastic in 30 (12.1 %) patients. According to AIDS Clinical Trials Group (ACTG) classification in 122 patients with AIDS-related Kaposi’s sarcoma, the stage at presentation was T1, I1, S1 in 56 (45.9 %); T1, I1, S0 in 32 (26.2 %); T0, I0, S1, in 10 (8.2 %) and T0, I0, S0 in 24 (19.6 %). Overall 92 (75.4 %) patients had poor prognosis disease comprising of 62 (81.6 %) females and 30 (65.2 %) males. However, there was no significant difference between CD4 counts in males and females (OR 1.54, 95 % CI 0.29–2.74, p = 0.675). Females had more disseminated cutaneous lesions involving an increased number of lesions at multiple anatomical sites compared with more localized lesions in the males (OR 1.9, 95 % CI 1.1–8.3, p = 0.001). Out of the 122 HIV patients, 25 (20.5 %) were on antiretroviral therapy at the time of diagnosis and their CD4+ count ranged from 156 to 812 cells/µl. Overall, CD4+ count available in 104 patients ranged from 34 to 798 cells/µl with a median of 148 (IQR = 140–152 cells/µl). A total of seventy-four HIV patients (71.2 %) had CD4+ count below 200 cells/μl and the remaining 30 patients (28.8 %) had CD4+ count of ≥200 cells/μ. Viral load among HIV positive patients was not determined in this study due to lack of facilities for this important work up.

**Table 1:** Anatomical site distribution (N = 387)

Anatomical site	Frequency	Percentages
Lower limb	112	28.9
Trunk	84	21.7
Oropharyngeal	54	14.0
Ocular	48	12.4
Upper limb	45	11.6
Lymph node	26	6.7
Viscera	12	3.1
Genitalia	4	1.0
Face	2	0.5

### Treatment modalities

The treatment modalities in this study included cytotoxic chemotherapy, radiotherapy, surgery and antiviral therapy (HAART) given either alone or in combination as shown in Table 2. Overall, 108 (43.5 %) patients were treated with both HAART and chemotherapy and 86 (34.6 %) patients were treated with chemotherapy alone. Of the 182 patients who had chemotherapy given either alone or in combination with HAART, only 76 (41.8 %) had completed the six cycles and the remaining 106 (58.2 %) patients did not complete their treatment cycles because of death in the course of treatment after two to five courses, treatment stopped because of severe side effects or were lost to follow up shortly after commencement of treatment. In this study chemotherapy was given either as Vincristine monotherapy or as a combination of Vincristine, doxorubicin and Bleomycin. The median time to start chemotherapy after the diagnosis of KS was 4 months (IQR = 2–6 months). Only 5 (2.0 %) patients received radiotherapy. Thirty-two HIV patients had only antiretroviral therapy. Excision of ulcerated lesion and limb amputation were performed in four (1.6 %) patients each respectively. The remaining 9 (3.8 %) patients had only supportive care because of poor Karnofsky performance score.

**Table 2:** Distribution of patients according to treatment modalities

Treatment modality	Number of patients	Percentages
Chemotherapy + ARV therapy (HAART)	108	43.5
Chemotherapy alone	86	34.6
ARV therapy alone	32	12.9
Radiotherapy alone	5	2.0
Excision of ulcerated lesions ± skin grafting/flap	4	1.6
Limb amputation	4	1.6
Supportive care only	9	3.6
Total	248	100

### Treatment outcome and follow up

A total of 66 treatment related complications were recorded in 64 patients giving a complication rate of 25.8 %. Table 3 below shows treatment related complications. These complications related to chemotherapy delayed chemotherapy by 1–2 weeks in 38 patients. Overall 126 (53.4 %) patients had significant improvement in quality of life (Karnofsky performance score ≥70) at the end of 1 year follow up. Sixty patients died at the end of 1 year giving a mortality rate of 24.2 %. Using multivariate logistic regression analysis, poor ACTG stage (OR = 4.5, 95 % CI (2.3–8.6), p = 0.011), CD4+ count <200 cells/µl (OR = 3.9, 95 % CI (1.1–5.8), p = 0.000), associated co-morbid illness (OR = 4.1, 95 % CI (2.2–7.2), p = 0.032), disseminated disease (OR = 6.7, 95 % CI (2.1–8.9), p = 0.001) and poor adherent to chemotherapy (OR = 7.3, 95 % CI (3.3–9.2), p = 0.021) were the significant independent factors associated with deaths. Patient’s follow-up ranged from 1 to 12 months. At the end of follow up period, out of the 188 survivors, only 62 (33.0 %) were available for follow up and the remaining 126 (67.0 %) were lost to follow up.

**Table 3:** Distribution of patients according to treatment related complications (N = 66)

Treatment related complications	Frequency	Percentages
Complications related to side effects of drugs/radiotherapy	54	81.8
Haematoxicity	18	
Digestive toxicity	16	
Peripheral neuropathy	13	
Hair loss	4	
Skin necrosis	1	
Others	2	
Complications related to surgical treatment	12	18.2
Surgical site infections	5	
Skin grafting failure	2	
Wound gaping/dehiscence	2	
Stump necrosis	2	
Phantom pain	1	

## Discussion

In this review, Kaposi’s sarcoma accounted for 2.4 % of all registered malignancies during the period of study. This figure is higher than the figure of 1.4 % reported by Mandong et al. [16] in Nigeria, but low compared to reports from central, east and southern Africa which was put at 1.5–12 % of all cancers [4, 5, 13, 14, 18–21]. In a previous study at the same centre by Chalya et al. [26], Kaposi’s sarcoma ranked second after malignant melanoma and accounted for 10.4 % of all skin cancers. This difference in the rate of Kaposi’s sarcoma reflects differences in the risk factors for Kaposi’s sarcoma from one country to another.

In the present study, AIDS-related Kaposi’s sarcoma was reported in 49.2 % of cases, a figure which is higher than 17–32 % reported from Central and Southern African countries [13–16]. AIDS-related Kaposi’s sarcoma has also been reported to be highly prevalent in Mozambique, a country that has one of the highest prevalence of HIV in the world [27]. In Western countries, the incidence of AIDS-related Kaposi’s sarcoma has dropped from about 15 % at the beginning of the AIDS outbreak to 0.3 % [28]. The high incidence of HHV-8 infection and limited access to HAART and other preventive measures explain in part the reason for increasing incidence of AIDS-related Kaposi’s sarcoma in sub-Saharan Africa [29].

In this study, Kaposi’s sarcoma was more prevalent in males than in females, with a male to female ratio of 1.4:1. This male predominance is in agreement with other many studies [15, 16]. However, in the current study AIDS related Kaposi’s sarcoma was predominantly a disease of females which is in keeping with others studies [30, 31]. The female predominance among AIDS-related Kaposi’s sarcoma in these studies may be attributed to the fact that the HIV epidemic is affecting an increasingly higher number of women in sub-Saharan Africa with over 60 % of persons living with the virus in this region being women. In addition, women are more frequently subjected to HIV testing as routine counseling and testing has been applied to perinatal settings, and this may have allowed for a greater number of AIDS-related Kaposi’s sarcoma cases to be identified among women [30, 31].

In keeping with the findings in other studies [15, 20, 32–36], females in this study reported to health facilities at a significantly younger age than males. This could indicate that females were infected with HIV at an earlier age or that immunosuppression from HIV infection and Kaposi’s sarcoma pathogenesis progresses more rapidly in females.

The finding that AIDS-related Kaposi’s sarcoma occurs at an earlier age when compared with patients without AIDS-related Kaposi’s sarcoma has been reported previously [1, 37, 38]. This is in keeping with our study where AIDS-related Kaposi’s sarcoma were found to be younger than those without AIDS-related Kaposi’s sarcoma This may be explained by the fact that HIV is reported to be most prevalent among those aged 20–40 years in sub-Saharan Africa, coupled with high-risk behavior and immunosuppression in this age group [1, 38].

Co-existing opportunistic infections, mainly TB, were a common feature in the recruited patients, with AIDS-related Kaposi’s sarcoma patients bearing a higher burden than patients without AIDS-related Kaposi’s sarcoma. Similar observation has been reported by other authors [12, 15, 20, 21]. This observation may be explained by the fact that the degree of immunosuppression is usually higher in patients with AIDS-related Kaposi’s sarcoma than in those without AIDS-related Kaposi’s sarcoma. HIV contributes to the pathogenesis of Kaposi’s sarcoma by inducing the immunosuppression necessary for the clinical expression of opportunistic disease. In addition, the HIV tat protein induces a number of cytokines known to promote HIV replication, while also inducing Kaposi’s sarcoma cell growth, invasion and angiogenesis [39–43].

Patients in this study presented with extensive and disseminated disease with 75.4 % having poor \ prognosis stage, a much higher proportion than reported from resource rich countries [44, 45]. Other reports from poor resource countries revealed that poor prognosis disease constitute 60–82 % of cases [14, 20, 36]. Majority of the patients with AIDS-related Kaposi’s sarcoma in this study presented with the disseminated form of the disease and most of them also had mixed lesions. AIDS-related Kaposi’s sarcoma usually follows an aggressive and fulminant course with involvement of multiple sites [20, 45]. In the present study female patients presented with more advanced AIDS-related Kaposi’s sarcoma, a finding that had been reported previously [20, 29, 46]. In addition, they had more aggressive disease which progressed rapidly. The reason why AIDS- related Kaposi’s sarcoma is more severe and progressed faster in females is not clear but does not seem to be related to immunologic differences since there was no significant difference between CD4 cells counts in males and females.

The lower limbs were most frequently affected with an associated lymphoedema which may be extensive and disproportionate to the extent of cutaneous disease. This lymphoedema may result from tumor involvement of dermal lymphatics or from the production by KS cells of growth factors that increase vascular permeability [46]. Additionally, HHV-8-induced exuberant proliferation of endothelial cells may lead to the occlusion of lymphatic vascular lumens leading to lymphoedema [46]. Why Kaposi’s sarcoma is common on the lower limbs is not clearly understood.

Involvement of the gastrointestinal tract (GIT) was seen in only 3.1 % of our patients. However, most patients with GIT Kaposi’s sarcoma are asymptomatic, and because the lesions are submucosal they are not visualized on contrast-enhanced radiographs [47]. Previous studies showed that asymptomatic GIT lesions have little clinical consequences hence; endoscopy should be carried out only on symptomatic patients [27, 47]. However, in this study, the number of GI lesions may be underestimated due to poor accessibility and affordability to endoscopic facilities.

In the present study, lymph node involvement by AIDS-related Kaposi’s sarcoma commonly occurred in adults whereas; in patients without AIDS-related Kaposi’s sarcoma lymph node involvement was exclusively a disease of children. This is in agreement with other researchers who reported similar observation [37, 48]. The fact that lymph node involvement by AIDS-related Kaposi’s sarcoma was seen in adults is contrary to earlier reports that lymph node involvement by Kaposi’s sarcoma is exclusively a disease of children [48]. This suggests that AIDS-associated KS presents atypically with lymph node, gastrointestinal tract disease and other organ involvement. Therefore the finding of atypical Kaposi’s sarcoma -lesions should arouse the suspicion of HIV infection.

As previously reported in other studies [49, 50], anaemia was a common presentation in patients with AIDS associated Kaposi’s sarcoma in this study. The causes of anaemia in AIDS-related Kaposi’s sarcoma are numerous, and may include viral infections, chronic diseases, abnormal alterations in the level of cytokines, drug therapies, opportunistic infections, and tumors infiltrating the bone marrow. Data from the Spectrum of Disease Study have indicated that correction of anaemia is associated with prolongation of survival in AIDS [51]. Thus efforts should be made to establish the cause of anaemia in AIDS-related KS and appropriate treatment instituted.

Several modalities of treatment have been used for Kaposi’s sarcoma including chemotherapy, radiation therapy, surgical excision and Highly Active Anti-Retroviral Therapy (HAART) in patients with AIDS-related Kaposi’s sarcoma [45]. The choice of treatment is determined by the stage of Kaposi’s sarcoma, its rate of progression, the degree of immune competence and HIV associated diseases [52]. The selection of therapy for Kaposi’s sarcoma must take into account the potential benefit and adverse effects of treatment, interactions with other medications, and potential impact on underlying immunosuppression [52]. A wide variety of chemotherapeutic agents, individually and in combination, have been evaluated for the treatment of Kaposi’s sarcoma. In high income countries, combination of vincristine, doxorubicin and bleomycin (VAB) that was considered the standard chemotherapy regimen for Kaposi’s sarcoma has been supplanted by liposomal anthracyclines due to their higher efficacy and reduced toxicity [44]. In addition, the angiogenic nature of Kaposi’s sarcoma makes it particularly suitable for therapies based on targeted agents such as metalloproteinase inhibitors, angiogenesis inhibitors and tyrosine kinase inhibitors [53]. In low income countries, the choice of therapeutic agents is limited to the combination of VAB or even more toxic drugs such as thalidomide because liposomal anthracyclines are not available or affordable [54]. In the present study, our patients were treated with a combination of vincristine, doxorubicin and bleomycin, similar to reports from other low income countries [54]. The internationally recommended chemotherapeutic agents such as Paclitaxel, Liposomal doxorubicin and immune modulators (alpha interferon) were not used in this study due to high cost and unavailability. However, in terms of cost effectiveness, the VAB regimen is the most rational treatment option for Kaposi’s sarcoma patients in poor resource settings [53, 54]. It has been reported that six cycles of this regimen could produce a significant and quick response in symptomatic patients with advanced KS and produce an overall response rate of 50–88 % [36, 54]. In our study, only 53.4 % of patients had significant improvement in quality of life at the end of the follow up period. This may be attributed to by the fact that more than a half of patients did not complete their treatment cycles because of death in the course of treatment after two to five courses, treatment stopped because of severe side effects or were lost to follow up shortly after commencement of treatment.

When dealing with localized bulky or cosmetically disturbing lesions, radiotherapy is the most effective local therapy. In this as in other reports, irradiated lesions regress with treatment, but recurrence, after 6 months is common [55]. In addition to providing effective palliation, radiotherapy is associated with minimal side effects. However, in our study, only 5 (2.0 %) of patients who required radiotherapy had access to this form of treatment. This concurs with other studies in resource-limited countries [20, 26]. Failure to access this modality of treatment in our patients can be explained by the fact that radiotherapy is not available in our center and therefore patients requiring this form of treatment had to travel long distances to receive radiotherapy at the oncological center. This sad observation calls for urgent establishment of radiotherapy services in our center.

Although surgery is effective in excision of localized isolated lesions, heroic surgery in the treatment of Kaposi’s sarcoma is unjustified and it is performed only in selected cases [20, 26]. In the present study, excision of ulcerated lesion and limb amputation were performed in four (1.6 %) patients each respectively. Similar surgical treatment pattern was performed by Ahmed and Muktar [29] in Nigeria.

Previous studies have shown that patients with AIDS-related Kaposi’s sarcoma are treated with a combination of chemotherapy and Highly Active Anti-Retroviral Therapy (HAART) with good results [29, 52, 54]. The effects of HAART on Kaposi’s sarcoma are multifactorial and include inhibition of HIV replication, diminished production of HIV-1 transactivating protein Tat, reconstitution of immune response against HHV-8 and possibly direct antiangiogenic activity by inclusion of protease inhibitors [56]. In the present study, decision to initiate systemic chemotherapy was based on the extent of Kaposi’s sarcoma in addition to other considerations such as patient KPS, end organ function, degree of immunosuppression, and other HIV co-morbidities. In patients with low tumor burden and slowly progressing disease, histological regression of existing KS lesions has been shown in response to HAART alone [56]. However, HAART alone cannot effectively control all cases of KS and there may be initial tumor progression as part of the immune reconstitution syndrome. In addition, it is not possible to state with certainty what proportion of patients with AIDS-related Kaposi’s sarcoma will benefit from HAART alone, or what are the precise characteristics that can be used to identify such patients. In low income countries like ours, chemotherapy consisting of a combination of vincristine, doxorubicin and bleomycin should be given simultaneously with HAART to patients that can physiologically withstand such therapy [55, 56]. The toxicities observed following chemotherapy in our patients are similar to those reported in other studies using same regimen and are well tolerated [57].

Palliative care for Kaposi’s sarcoma patients who are well tolerated may include adequate pain relief, reduction of the size of tumors with radiotherapy and reduction of the offensive smell of ulcerated lesions with appropriate dressing argent. Prevention and treatment of other opportunistic infections is necessary as uncontrolled infections may stimulate Kaposi’s sarcoma progression probably due to production of angiogenic cytokines [29]. In our series, nine (3/8 %) of patients had only supportive care because of poor Karnofsky performance score and this included adequate pain relief by analgesics, reduction of offensive smell of ulcerated lesions with appropriate dressing argent and adequate treatment of other opportunistic infections.

One year mortality at our facility was 24.2 % among the-KS patients. This figure is high compared to 22.9 % that was reported by Ahmed and Muktar [29] in Nigeria. This high rate may be due not only to the fact that most of our patients presented with advanced HIV disease but also to the unavailability of chemotherapy and radiotherapy. In this study, poor ACTG stage, CD4+ count <200 cells/µl, associated co-morbid illness, disseminated disease and poor adherent to chemotherapy were the significant independent factors associated with mortality in multivariate logistic regression analysis.

The follow-up of patients in this study was generally poor, and data on long-term survival were not available. This observation concurs with Asuquo et al. [37] in Nigeria. Poor follow-up of patients in our study may be explained by the fact that the majority of patients were lost to follow-up at the end of follow up period. In this study, the facility for measuring the viral load and differences in viral genome was not available in our centre; this could have explained the differences in response to chemotherapy in the HIV subset. Radiation therapy provides excellent results with few side effects in all types of Kaposi’s sarcoma. However, only a few of our patients had radiotherapy, as this facility is not available in our centre. Those referred for radiotherapy were unwilling because of cost and long distance. However, despite these challenges, the study has provided local data that can help healthcare providers in the management of patients with Kaposi’s sarcoma. The challenges identified in the management of Kaposi’s sarcoma in our setting need to be addressed in order to deliver optimal care for these patients.

## Conclusion

Kaposi’s sarcoma remains a common malignant vascular tumor in this region and is commonly associated with HIV/AIDS. The patients present late with extensive and advanced disease that requires systemic treatment. Late presentation, Lack of awareness of the disease, poor accessibility to healthcare facilities, poor diagnostic and treatment resources, irregular availability of chemotherapy and HAART, the high cost of care, loss to follow up and lack of radiotherapy services at our centre are among the hallmarks of the disease in this region and pose a great challenge in the management of these patients. Therefore public enlightenment, early diagnosis, and effective cost-effective treatment and follow-up will help reverse this trend.

## Abbreviations

ACTG: AIDS Clinical Trials Group
AIDS: acquired immunodeficiency states
AIDS-KS: AIDS-related Kaposi sarcoma
BMC: Bugando Medical Centre
CTC: care and Treatment Clinic
CUHAS: Catholic University of Health and Allied Sciences
GIT: gastrointestinal tract
HAART: highly active antiretroviral therapy
HHV-8: human herpes virus-8
HIV: human immunodeficiency virus
IQR: interquartile range
KS: Kaposi’s sarcoma
KPS: Karnofsky performance score
SPSS: statistical package for social sciences
VAB: vincristine, doxorubicin and bleomycin
